# An Unusual Schwannoma in the Proximal Forearm: A Case Report

**DOI:** 10.7759/cureus.6231

**Published:** 2019-11-25

**Authors:** Tayfun Hakan, Yusuf Kılıç, Erhan Çelikoğlu, Süheyla Ekemen

**Affiliations:** 1 Neurosurgery, Private Practice, Intermed Çiftehavuzlar Outpatient Clinics, İstanbul, TUR; 2 Surgery, Intermed Çiftehavuzlar Outpatient Clinics, İstanbul, TUR; 3 Neurosurgery, Fatih Sultan Mehmet Teaching and Research Hospital, İstanbul, TUR; 4 Pathology, Bahçeşehir University, Faculty of Medicine, İstanbul, TUR

**Keywords:** forearm, median nerve, neurilemmoma, peripheral nervous tumor, schwannoma, enucleation, microsurgery, benign

## Abstract

Schwannomas are common, well-encapsulated benign tumors of the peripheral nerves. They rarely emerge from the median nerve in the forearm. Here we report a case of an unusual schwannoma measuring 3 × 4 × 3 cm originating from the median nerve in the proximal forearm of a 49-year-old man. The mass was painless, but Tinel’s sign was positive. Ultrasonography showed a solid, hypoechoic mass with central cystic areas in the flexor muscle group in the left forearm. Magnetic resonance imaging with contrast enhancement in T1 sequences revealed that it originated from the median nerve. Surgical resection was performed by separating the nerve fibers from the tumor without any complications. Histological examination confirmed it as a schwannoma.

## Introduction

Schwannomas, which develop from Schwann cells, are the most common benign tumors of the peripheral nerves [1], accounting for 90% of all peripheral neural tumors and less than 8% of all benign soft tissue tumors [2]. Schwannomas typically involve the ulnar nerve; less than 7% of these tumors are located along the median nerve sheath in the upper extremities [3]. Schwannomas are typically solitary tumors ranging from 1.5 to 3 cm in diameter, and large tumors are rare [4].

The clinical symptoms and radiological images of schwannomas can be similar to the other soft tissue tumors. Intraoperative findings and microscopic analyses with immunohistochemistry features make it easy to differentiate schwannomas from other tumors. Histologically, schwannomas have two components, namely highly cellular Antoni A and loosely cellular Antoni B patterns, and show uniformly intense immunostaining for S-100 protein [5].

Here, we report a rare schwannoma in a 49-year-old male patient and present the clinical, radiological, and histological findings.

## Case presentation

**Figure 1:**
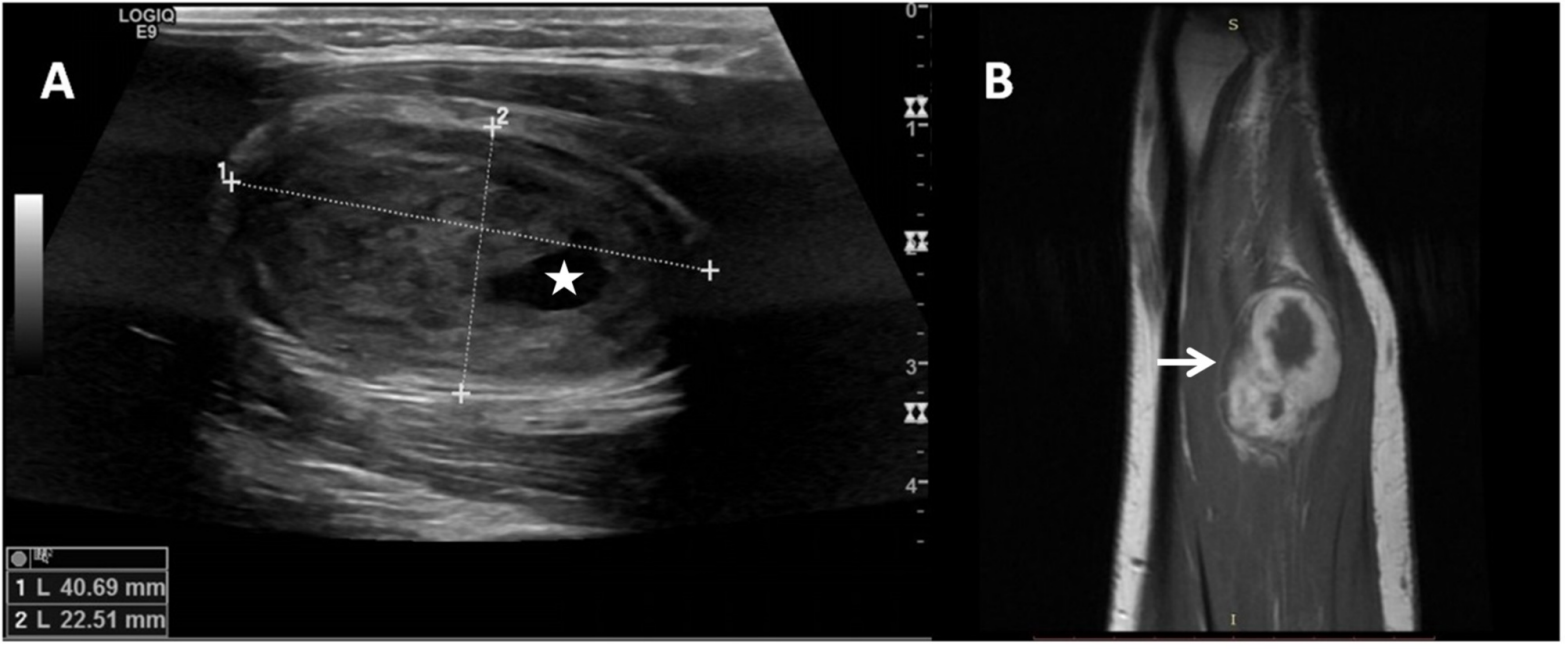
Ultrasonography and magnetic resonance images of the tumor (A) Ultrasonography revealed a hypoechoic solid mass with central cystic clarity (asterisk) in the proximal forearm. (B) Magnetic resonance imaging with contrast enhancement revealed a soft tissue mass (white arrow) on the median nerve route in the flexor muscles of the forearm.

A 49-year-old man presented with painless swelling in his left arm. A round, hard, mobile mass was detected at the volar aspect of his left proximal forearm on physical examination. Tinel’s sign was positive. No neurological deficits were detected. The patient had no comorbidities and no remarkable medical or surgical history. His general condition was optimal. Ultrasonography (USG) showed a solid, hypoechoic mass with central cystic areas located in the flexor muscles group in the left forearm. Magnetic resonance imaging (MRI) revealed a huge, well-encapsulated mass originating from the median nerve on contrast-enhanced T1 sequences (Figure 1A, 1B).

**Figure 2:**
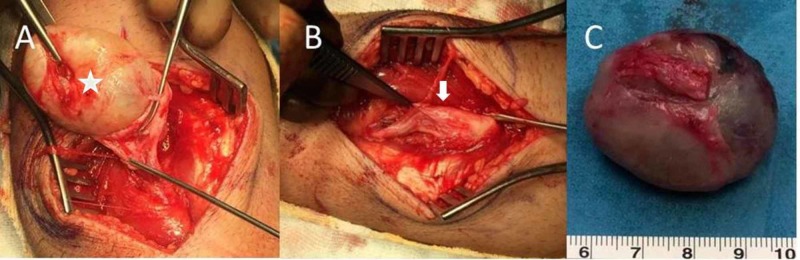
Intraoperative images of the tumor (A) Intraoperative image of the tumor (asterisk) originating from the left median nerve in the proximal forearm. (B) Image of the median nerve (white arrow) following tumor resection. (C) Completely resected tumor.

USG-guided biopsy under local anesthesia confirmed the diagnosis of a schwannoma. The patient underwent surgery under local anesthesia for excision of the tumor. A tumor-centered incision was made at the midline of the ventral face of the left mid forearm for adequate exposure of both the proximal and distal sides of the tumor on the median nerve. A round, yellow-pink tumor was enveloped in an eccentric true capsule between the brachioradialis and flexor carpi radialis muscles under the skin. Following the incision of the tumor capsule, the nerve fibers entering and exiting the tumor were isolated. The encapsulated 3 × 4 × 3 cm sized mass of median nerve at the proximal forearm was completely resected in one piece without nerve injury (Figure 2).

**Figure 3:**
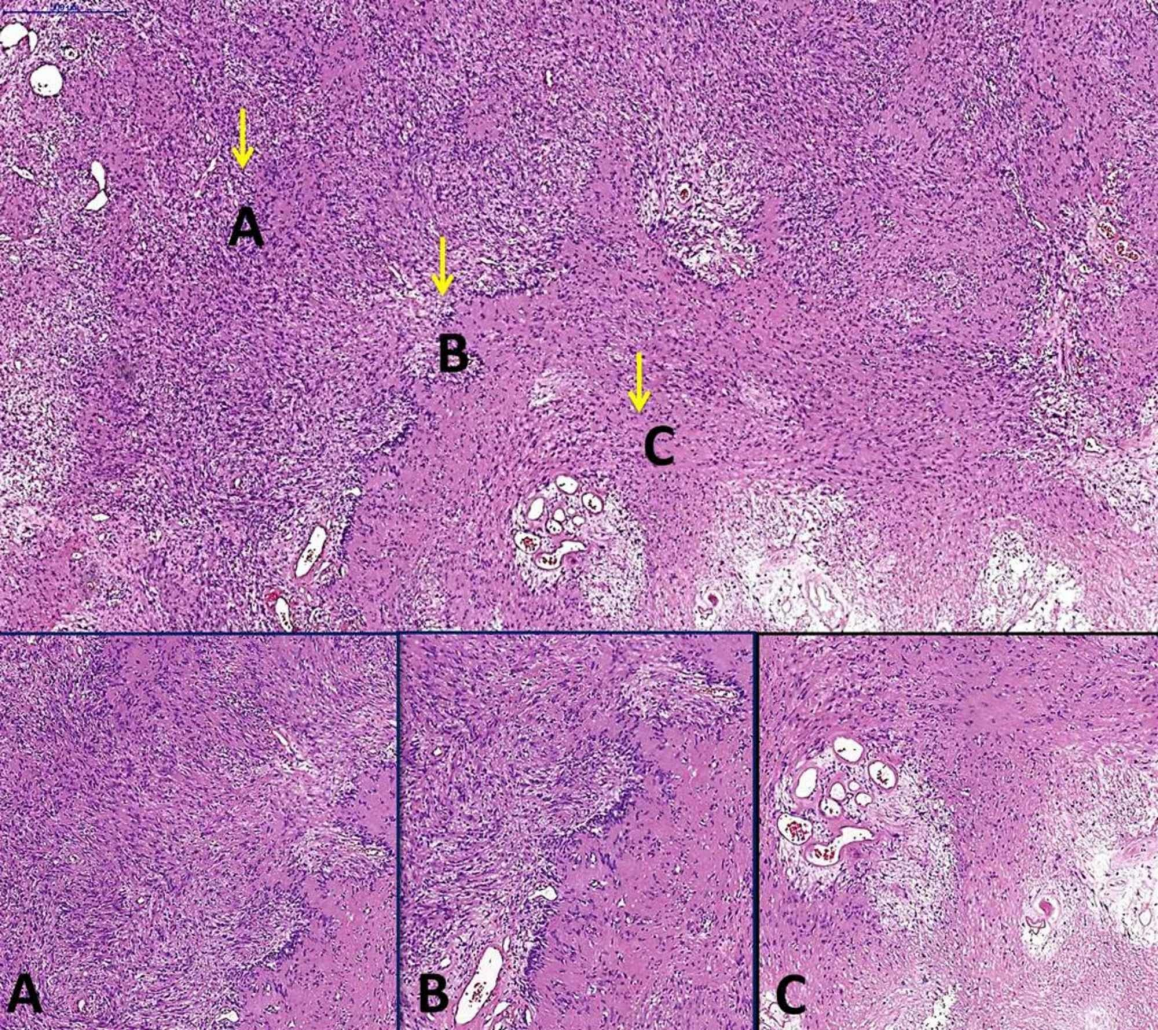
Pathological images of the tumor Big figure:  Biphasic tumor, H&E 5x (Case Viewer 3DHISTECH). Little figures H&E 10X (Case Viewer 3DHISTECH). (A) Compact hypercellular Antoni A areas. (B) Nuclear palisading around fibrillary process (Verocay bodies). (C) Myxoid hypocellular Antoni B areas.

Tinel’s sign of the median nerve was completely resolved in the early postoperative period. The pathological diagnosis was determined to be a schwannoma (Figure 3).

**Figure 4:**
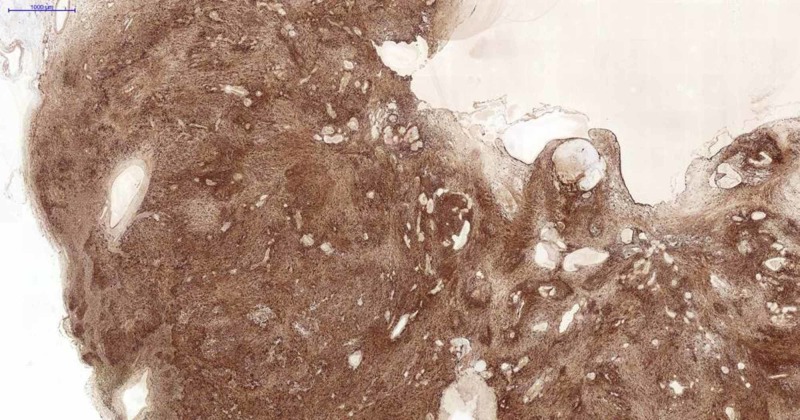
Immunohistochemical staining images of the tumor Schwannoma S-100 immunohistochemical staining ×2.

Immunohistochemical analysis of the tumor showed strong, diffuse expression of S100 protein (Figure 4).

The Ki-67 [DAKO (MIB-1)] index was 2%-3%, and smooth muscle actin (SMA) [SCYTEK (1A4)] was negative.

## Discussion

Schwannomas, also known as neurilemmoma, are common, soft tissue tumors of the extremities that can be categorized as proximal i.e., those on the brachial plexus and upper arm, or distal i.e., those on the forearm and hand [6]. Schwannomas emerge from nerves covered with Schwann cell sheaths and are surrounded by true capsules consisting of epineurium.

Schwannomas may be found incidentally as painless masses or can exhibit neurological symptoms due to the compression of the surrounding structures. Symptomatic tumors can present with neurogenic pain in the extremities, local swelling, paresthesia, and motor weakness [7]. A positive Tinel’s sign carries a high predictive value as in this present study.

Accurate diagnosis is the first step for the treatment of schwannomas. USG can identify the localization and origin of the nerve tumor and show its relationship with non-involved nerve fibers [1]. MRI also plays an important role in preoperative diagnosis; schwannomas show intermediate intensity to muscles in T1-weighted images and are hyperintense in T2-weighted images [8]. Uniform enhancement is usual for schwannomas, although more heterogeneous enhancement may be seen in larger tumors such as the one in the present study. A post-contrast T1-weighted sequence can show the tumor from the surrounding fascicles of the nerve. Preoperative biopsy is recommended to plan the treatment strategy in cases where a malignant peripheral nerve sheath tumor is suspected [7]. For an experienced surgeon, preoperative biopsy under the guidance of high-resolution ultrasound imaging combined with MRI can be safely performed [1]. Although USG-guided biopsy seems to be less invasive for soft tissue tumors, the high risk of iatrogenic damage to the fascicles and hemorrhage within the tumor should be considered [7]. Patients often experience worsening of neurogenic pain and development of neurological deficits. In the present study, ultrasound-guided biopsy was performed without any iatrogenic complications.

Microsurgical resection is recommended for the treatment of all symptomatic schwannomas at first diagnosis and for asymptomatic cases with MRI evidence of increasing tumor size [1,9]. Neurological complications, such as sensory or motor deficits or neuropathic pain, may arise following schwannoma enucleation [1,4,6]. A careless puncture or unplanned resection of the tumor may cause postoperative neurological deficits. A true capsule consisting of epineurium may allow the enucleation of schwannomas without nerve damage. Complete resection should be attempted for better prognosis in terms of functional outcome and overall survival and to reduce the risk of tumor recurrence [9].

Neurofibroma and malignant peripheral nerve sheath tumors are differential diagnoses of Schwann cell neoplasms. Pathological and immunophenotypic features are useful for this differentiation [5]. The typically diffuse, strong expression of S100 protein, low Ki-67 (2%-3%) index, and negative actin (SMA) helped diagnose the schwannoma in this present study.

## Conclusions

Schwannomas originating from the median nerve in the proximal forearm are rare. USG and MRI features are very useful for preoperative diagnosis. Pathological and immunophenotypic features are helpful for differential diagnosis of the schwannomas. Complete enucleation of large schwannomas is possible with adequate microsurgical techniques without any complication.
